# C3aR plays both sides in regulating resistance to bacterial infections

**DOI:** 10.1371/journal.ppat.1010657

**Published:** 2022-08-04

**Authors:** Jesse A. Corcoran, Brooke A. Napier

**Affiliations:** Department of Biology and Center for Life in Extreme Environments, Portland State University, Portland, Oregon, United States of America; Geisel School of Medicine at Dartmouth, UNITED STATES

## Abstract

Activation of the complement pathway results in the production of bioactive C3a, a product of C3 cleavage, which interacts with membrane-bound receptor C3aR to regulate innate immune cell function and outcome of bacterial infection. Specifically, previous research has identified mechanistically distinct and cell type–specific roles for C3aR in regulating innate immune cell inflammatory state, antimicrobial killing capacity, and metabolism. Historically, the production of C3a has been relegated to the serum; however, recent studies have provided evidence that various cell types can produce intracellular C3a that stimulates intracellular C3aR. In light of these new results, it is imperative that we revisit previous studies regarding the role of C3aR in controlling bacterial infections and analyze these results in the context of both extracellular and intracellular C3a production and C3aR activation. Thus, this review will cover specific roles of C3aR in driving cell type–specific and tissue specific responses during bacterial infections and emphasize the contribution of the C3a–C3aR axis in regulating host resistance to bacterial infection.

## Introduction

The complement system plays an essential role in defense against bacterial pathogens, and recent insights into the role of tissue-specific and cell-autonomous complement-mediated immunity have inspired a systemic review of complement literature in the context of these new functions. Specifically, many of the complement pathway effector functions are downstream of the cleavage of complement factor 3 (C3) into biologically active C3a and C3b. C3b acts as an opsonin and activates the lytic pathway. C3a is a soluble factor that activates its cognate receptor C3aR in an autocrine and paracrine fashion on innate immune cells to regulate local and systemic inflammation.

C3aR belongs to the family of inhibitory G protein–coupled 7 transmembrane-containing receptors (G_i_) and is expressed both intracellularly and extracellularly on a wide variety of cells [1]. Activation of membrane-bound G_i_ proteins lead to the release of the α subunit, which suppresses production of intracellular cAMP via inhibition of adenylyl cyclase, and β/γ subunits, which have been shown to regulate ERK1/2 and JNK activation through PI3K/AKT activity in a cell type–specific manner [2]. Further, it has been shown that C3aR undergoes rapid (approximately 15 seconds) inactivation via GPCR kinase (GRK)-dependent phosphorylation in mast cells [3,4] and internalization within granulocytes and epithelial cells [5,6]. Studies that have characterized C3aR as a G_i_ within innate immune cells are very limited and the effect C3aR activation within innate immune cells on downstream signaling cascades has not been fully defined.

It has been reported in murine dendritic cells (DCs) that intracellular production of cAMP is reduced following stimulation with C3a, with an increase in phosphorylation of the PI3K/AKT and ERK pathways [7]. Similarly, human DCs showed reduced intracellular cAMP production and increased PI3K/AKT, ERK, and NF-κB signaling following stimulation with C3a [8]. Further, murine DCs pretreated with a C3aR agonist saw reduced p38 MAP kinase phosphorylation [9]. In human monocytes and macrophages, C3aR activation leads to enhancement of IL-1β production that is regulated by enhanced ERK1/2 phosphorylation [10]. Further, we have shown blockade of C3aR activation in primary murine and human macrophages using the nonpeptide inhibitor SB290157 leads to reduced TNF and IL-6 secretion and p38 MAP kinase phosphorylation during LPS or IFNβ stimulation [11]. Lastly, in a murine model of spinal cord injury, activation of C3aR by C3a led to increased ERK1/2 phosphorylation in bone marrow–derived neutrophils [12]. These data suggest that C3aR is acting as a canonical G_i_ within innate immune cells.

Importantly, some G_i_ proteins can alter Ca^2+^ mobilization. It was found that treating human neutrophils and monocytes with nanomolar concentrations of exogenous C3a triggered the rapid influx of Ca^2+^ from the extracellular medium [13,14]. Further, mouse peritoneal macrophages treated with a peptide C3a agonist induced a significant influx of intracellular Ca^2+^ that was dependent on C3aR [15]. Together, these data partially define the role of C3aR activation in innate immune cell signaling and suggest that C3aR may play a cell type–specific role in regulation of cAMP, PI3K/AKT-dependent signaling cascades, and Ca^2+^ mobilization. However, all of these studies have assumed that C3aR is located at the plasma membrane and have only analyzed cytoplasmic changes in signaling cascades and secondary messenger molecules, potentially missing important organelle-specific roles of C3aR activation within innate immune cells.

To this point, it was originally thought that production of C3a occurred exclusively within the serum by C3 convertases; however, recent publications have elegantly shown intraphagosomal production of C3a by Cathepsin L and intracellular activation of C3aR within multiple cell types [16–19]. Liszewski and colleagues have shown that in naive T cells, activation of phagosomal C3aR by C3a leads to induction of mTOR activation required for tonic metabolic requirements [18]. This was the first indication that C3aR may be regulating metabolism directly. Further, it was recently shown that in epithelial cells, C3aR is endocytosed upon oxidative stress and is trafficked to the outer mitochondrial membrane [6]. In these studies, activation of C3aR on the mitochondrial membrane increased Ca^2+^ uptake, which inhibited mitochondrial respiration via blocking state III ADP-driven respiration [6]. These new results have provided a novel paradigm that can now be used as a guide for interpreting past and future findings on C3aR function within the innate immune system.

Importantly, C3aR activation results in pathophysiological changes seen in infection models using both gram-negative and gram-positive bacteria, with both intracellular and/or extracellular life cycles. Surprisingly, little molecular detail underlying C3aR-dependent control of innate immunity is understood. However, many infection models using global C3aR knockout mice (C3aR^−/−^) have provided exciting insights into the dynamic orchestration of inflammation and infection outcome by C3aR. Here we discuss activation of C3aR during bacterial infection leading to cell type–specific pleiotropic effects, including modulation of signaling pathways and release of cytokines, reprogramming of innate immune cell function, enhanced inflammasome activation, and recruitment of inflammatory cells to infected tissues. Further, we examine how C3aR contributes to the complement system’s role in driving innate immune defenses and host resistance to bacterial infection.

### C3aR reprograms neutrophils during systemic and local bacterial infection

Neutrophils are innate effector cells that are rapidly recruited to the sites of infection and are important in the acute phase of bacterial infection. Neutrophils can promote bacterial resistance by clearing bacterial infections, while conversely, they can promote bacterial persistence by inducing tissue damage [20]. C3aR is expressed by neutrophils in the blood, and some reports suggest that serum C3a can enhance neutrophil antimicrobial function and act as a chemoattractant; however, the role of C3aR in neutrophils is currently under debate [21]. Here, we highlight the double-edged sword of enhancing neutrophil recruitment and activation by C3aR during bacterial infection.

#### C3aR reprograms neutrophils during systemic *Neisseria meningitidis* infection

*Neisseria meningitidis* (meningococcus) is a gram-negative bacterial pathogen that often asymptomatically inhabits the human nasopharynx, yet can cause invasive meningococcal disease (IMD) in susceptible individuals (infants, the elderly, and the immunocompromised) [22]. IMD manifests in meningitis and septicemia that can result in neurological disorders. Upon acquisition of *N*. *meningitidis* through inhalation, the organism attaches to and invades the mucosal epithelium in the upper respiratory tract [20]. Mucosal epithelial cells detect *N*. *meningitidis* via TLR2, TLR4, and NOD1 to induce production of IL-8, IL-6, TNF, and other inflammatory cytokines [20]. In susceptible individuals, *N*. *meningitidis* can evade killing by the mucosal immune response, entering the blood to survive and rapidly replicate [22]. Importantly, infection in the blood stimulates massive recruitment of neutrophils, which is both helpful for clearance and detrimental in causing host tissue damage associated with IMD [20].

**Fig 1:**
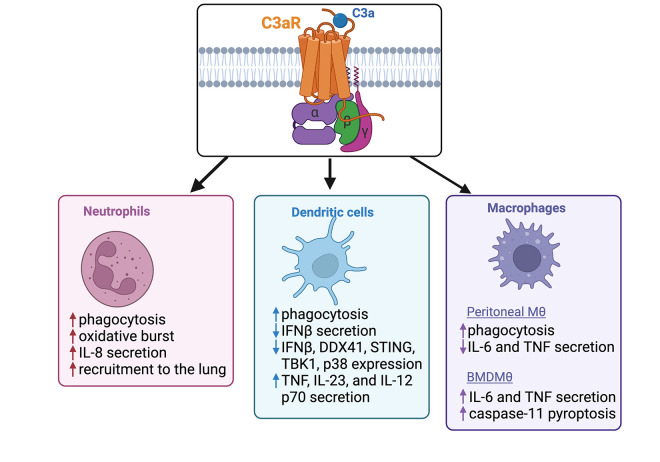
Schematic of C3aR-dependent innate immune cell function during bacterial infections.

In a murine sepsis model of *N*. *meningitidis*, it was found that C3aR^−/−^ mice displayed enhanced disease severity, increased colony-forming unit (CFU) in the blood, and a decrease in survival, compared to wild-type (WT) mice [23]. Considering the important role neutrophils play in driving the response to *N*. *meningitidis*, they measured the antimicrobial capacity of neutrophil intrinsic C3aR by pretreating whole human blood with a C3aR antagonist (C3aRi; SB290157) and infecting with *N*. *meningitidis*, which resulted in decreased secretion of IL-8 and significant inhibition of oxidative burst and phagocytosis (Fig 1) [23]. These data suggest that the protective effect of C3aR during *N*. *meningitidis* infection may be in part explained by its driving enhanced antimicrobial neutrophil activity. Additionally, this group found that early in infection, there was decreased IL-6 in the blood of C3aR^−/−^ mice [23]. Immune cells detect *N*. *meningitidis* via TLR2, TLR4, and NOD1 to induce production of inflammatory cytokines [20] and C3aR has been shown to enhance production of inflammatory cytokines downstream of TLR2 and TLR4 activation within macrophages [11]. C3aR may similarly be enhancing inflammation downstream of TLR activation within neutrophils, although this result has not been shown. Future molecular studies understanding the C3aR-dependent signaling cascades required for modulating neutrophil function and enhancing systemic production of IL-6 may provide insight into the protective role of C3aR in *N*. *meningitidis* infection.

#### C3aR recruits neutrophils to the lung and promotes *Pseudomonas aeruginosa* infection

*Pseudomonas aeruginosa* is a gram-negative pathogen that is often found in the environment and is a major cause of healthcare-associated infections, including pneumonia and infections involving the urinary tract, wounds, burns, and the bloodstream [24]. Colonization of the respiratory tract by *P*. *aeruginosa* results in localized inflammation driven by host cell production of cytokines and chemokines, and the subsequent recruitment of neutrophils to the lung. Various *P*. *aeruginosa* virulence components (ex: LPS, type III secretion system products, and pili) induce production of inflammatory cytokines by activating pattern recognition receptors (PRRs; typically, TLR4 and TLR5), reviewed elsewhere [25,26].

Numerous studies have worked to demonstrate the role of C3aR and C3a in driving acute inflammation in pulmonary allergy, but not lung infection. Thus, in 2006, Mueller-Ortiz and colleagues modeled *P*. *aeruginosa* pulmonary infection via intranasal infection and found C3aR^−/−^ mice displayed decreased recruitment of neutrophils to the lungs and increased bacterial clearance in the blood and lungs of mice, compared to WT mice [27]. These data are the first to suggest that presence of C3aR is deleterious during pulmonary bacterial infection. Furthermore, the C3aR^−/−^ mice showed decreased inflammatory cytokine (IL-6, TNF, and IL-1β) secretion in the bronchoalveolar lavage (BAL) [27]. Interestingly, mice deficient in the TNF receptor [28], IL-1 receptor [29], IFNγ receptor [30], or IL-18 [31] share similar phenotypes as the C3aR^−/−^ mice: less inflammation and decreased neutrophil recruitment to the lungs. It is now appreciated that inflammatory cytokines likely impair bacterial clearance from the pulmonary compartment and enhance tissue damage during *P*. *aeruginosa* infection and that C3aR contributes to this damage.

Additionally, these studies correlate with our current knowledge on the function of C3aR in amplifying inflammatory cytokine response during bacterial infection. Napier and colleagues found that C3aR is required for p38 MAP kinase phosphorylation downstream of TLR4 activation by LPS [11]. Recently, Coates and colleagues found that inhibiting p38 MAP kinase activity during *P*. *aeruginosa* infection of bronchial epithelial cells depleted IL-6 and CXCL8 production [32]. These findings bolster the hypothesis that C3aR may be enhancing PRR detection of *P*. *aeruginosa* and subsequent release of inflammatory cytokines during infection via the p38 MAP kinase pathway in the lung. Further studies will need to be conducted to identify whether neutrophil-intrinsic C3aR activation or bystander C3aR activation is leading to cytokine secretion that regulates neutrophil chemotaxis to the BAL during *P*. *aeruginosa* infection of the lung.

### C3aR mediates DC inflammatory states to enhance bacterial resistance

DCs play an imperative role in the innate immune response by enhancing inflammation and inducing adaptive immune responses that are critical for clearance of bacteria during infection. The recent development of a C3aR reporter mouse has shown C3aR is expressed in bone marrow–derived dendritic cells (BMDCs), lung-resident CD11b+ conventional DCs (cDCs), and monocyte-derived DCs [1], demonstrating that C3aR is widely expressed on various DC subsets. Here, we review possible mechanisms by which C3aR regulates resistance to 2 bacterial pathogens via dampening expression of type I interferons and enhancing inflammatory cytokines within DCs.

#### C3aR shields against *Listeria monocytogenes*

*Listeria monocytogenes* (LM) is a gram-positive facultative intracellular pathogen transmitted via contaminated food and can lead to sepsis and meningitis [33]. After ingestion of contaminated food, LM enters intestinal epithelial cells where it replicates and disseminates via the lymph and blood to target organs (liver and spleen) [33]. Early resistance to LM is dependent on NF-κB and IRF3/7 activation, subsequent production of inflammatory cytokines (TNF, type I-III interferons, etc.), and the recruitment of activated monocytes, macrophages, and neutrophils to the sites of infection [33]. Clearance of infection is typically dependent on CD8^+^ and CD4^+^ T-cell responses [33,34]. Additionally, LM induces apoptosis in macrophages, neutrophils, and DCs, which are all important for T cell–mediated immunity and effective clearance of infection [35,36].

To understand the role of C3aR in driving LM infection dynamics, Mueller-Ortiz and colleagues modeled LM infection within C3aR^−/−^ mice and showed a 50% decrease in survival and more severe systemic disease, compared to WT mice [34]. This decrease in survival of C3aR^−/−^ mice correlated with an increase in bacterial burden within the spleen and liver and enhanced production of IFNγ, TNF, and IL-6 in the blood [34]. These results suggest the presence of C3aR acts to reprogram the host inflammatory response to LM and enhance bacterial resistance. However, which cell type that requires C3aR in order to enhance bacterial clearance remains unclear. It is also unknown whether C3aR activation is dampening inflammation directly, or if there are higher concentrations of inflammatory cytokines in the blood of C3aR^−/−^ mice because they have larger bacterial burdens in the liver and spleen.

This same group found that the anaphylatoxin receptor C5aR1, which detects the bioactive C5a molecule, protected against LM infection by dampening type I IFN expression (IFNα/β) [37]. Type I IFNs are induced by cyclic di-AMP from LM stimulating DDX41 and STING, which lead to sensitization of infected cells to apoptosis [9]. There was no report on the effect of C3aR on type I IFNs in their 2013 report; however, a follow-up study from this lab showed that treatment of primary BMDCs with C3a followed by challenge with cyclic-di-AMP or infection with LM resulted in decreased expression and production of the type I IFN, IFNβ [9]. Additionally, they found secretion of inflammatory cytokines TNF and IL-6 were not affected by C3a pretreatment in BMDCs [9]. When LM-derived cyclic di-AMP is detected by DDX41, it complexes with STING and signals to TBK1 and p38 MAPK to induce the type I IFN response [38]. They found pretreatment with an C3aR agonist followed by a cyclic di-AMP challenge reduced expression of key mediators of the type I IFN response (DDX41, STING, TBK1, and p38) [9]. These data suggest that C3aR activation within BMDCs inhibits the expression of key type I IFN response regulators through an unknown mechanism, thus resulting in decreased expression of IFNβ during subsequent challenge with LM (Fig 1). They conclude that this C3aR-dependent decrease in IFNβ may contribute to enhanced survival and lower bacterial burden in WT mice, compared to C3aR^−/−^ mice infected with LM. Future studies using adoptive transfer of C3aR^−/−^ BMDCs into irradiated WT mice will be key in understanding if the DC-intrinsic role of C3aR impacts the host inflammatory state and bacterial resistance to LM.

#### C3aR makes DCs hungry for *Porphyromonas gingivalis*

*Porphyromonas gingivalis* is a gram-negative anaerobic oral bacterium that persists within periodontal pockets and is a key pathogen associated with human periodontitis [39]. Periodontitis is characterized by inflammatory damage of the gums and periodontal support tissues [40]. *P*. *gingivalis*–dependent activation of TLR2-phosphoinositide 3 kinase (PI3K) pathway can increase destruction of periodontal tissues and trigger inflammation necessary for further invasiveness [39,40]. Interestingly, TLR polymorphisms are associated with susceptibility of adolescents to periodontitis [41], demonstrating the importance of TLR-mediated innate immune responses in driving *P*. *gingivalis* resistance. Typically, *P*. *gingivalis* orchestrates inflammatory disease by inducing dysbiosis in the oral microbiome [39]; however, it can also invade and replicate within multiple cell types, including neutrophils, macrophages, and DCs [40].

In a study identifying the requirement for C3aR in the invasion and replication of *P*. *gingivalis* within DCs, researchers found WT BMDCs—but not C3aR^−/−^ BMDCs—treated simultaneously with C3a and infected with *P*. *gingivalis* showed enhanced bacterial clearance [40]. Further, BMDCs treated with C3a showed enhanced phagocytosis of *P*. *gingivalis* and increased secretion of TNF, IL-23, and IL-12 p70 (Fig 1) [39]. These data demonstrate that the C3a–C3aR axis is working to enhance clearance of bacteria and the production of inflammatory cytokines in response to *P*. *gingivalis* infection within BMDCs. Interestingly, they found that BMDCs from TLR2^−/−^ mice showed a nearly identical increase in *P*. *gingivalis* CFU as the C3aR^−/−^ BMDCs [39]. It has been previously shown that when *P*. *gingivalis* enters BMDCs, it uses its Mfa1 fimbriae to interact with DC-SIGN, which directs the bacterium into intracellular vesicles that escape autophagosomal degradation [42]. TLR2 can block the subversion of *P*. *gingivalis* autophagosomal degradation in BMDCs, thus enhancing intracellular killing [43]. Currently, there are no studies that identify the potential mechanism of the C3a–C3aR axis enhancing TLR2-dependent autophagosomal degradation of *P*. *gingivalis* in BMDCs. Importantly, it had been previously shown that macrophages treated with C3a and infected with *P*. *gingivalis* did not have any effect on bacterial burden [44]; however, this study did not investigate the role of phagosomal C3a or the necessity of C3aR activation for *P*. *gingivalis* survival within macrophages. Thus, these data show that C3aR-dependent intracellular killing of *P*. *gingivalis* is cell type-specific and underline potential differences between inflammatory signaling pathways between cell types [44]. It’s important to note that *in vitro* studies using C3aR^−/−^ BMDCs may not reflect the phenotypes of cells *in vivo* and highlights the need to better understand the contributions of different cell types during *P*. *gingivalis* infection *in vivo*. Further, these studies highlight the lack of known mechanisms driving C3aR-dependent intracellular *P*. *gingivalis* killing and leave open the possibility that C3aR-enhanced TLR2 signaling may be driving this resistance to bacterial infection.

### C3aR dampens macrophage function during Uropathogenic *Escherichia coli* (UPEC) infection

Tissue-resident macrophages are sentinel immune cells critically involved in defending the host from bacterial infection via phagocytosis and destruction of the pathogen and induction of inflammatory cytokines. We have previously shown that macrophage-intrinsic C3aR enhances induction of TLR4-dependent inflammatory cytokines and activation of the caspase-11 inflammasome during *Salmonella typhimurium* and *Shigella flexneri* infection, demonstrating a macrophage-intrinsic mechanistic link between C3aR activation and TLR activation [11]. It is currently unclear if macrophage-intrinsic C3aR regulates *in vivo* bacterial infection. Here, we will review the one study that defines a role of C3aR in controlling UPEC infection by manipulating macrophage function.

#### C3aR within macrophages destroys tissues during UPEC infection

Uropathogenic *E*. *coli* (UPEC) is a gram-negative bacterial pathogen and accounts for roughly 80% of urinary tract infections (UTIs), which can lead to cystitis in the bladder and acute pyelonephritis in the kidneys [45]. Once in the urinary tract, UPEC gains access to superficial bladder epithelial cells (also called facet or umbrella cells) that provides protection from phagocytes [46]. As UPEC replicates intracellularly, it actively suppresses the host inflammatory response, influx of neutrophils, and production of antimicrobial peptides (ex: defensins) [46]. The production of inflammatory cytokines (IL-6, IL-8, and IL-1β) and the neutrophilic response during UPEC infection are critical for host clearance of infection; however, an excessive inflammatory response to UPEC infection can result in deleterious renal pathology [45,46].

To evaluate the role of C3aR in UPEC pathogenesis, Wu and colleagues used a well-established murine model of ascending UTI that leads to pyelonephritis. They found that C3aR^−/−^ mice displayed impaired bacterial clearance in the kidney and more-severe kidney damage, compared to WT mice [45]. They demonstrated that the transfer of WT bone marrow into C3aR^−/−^ mice subsequently infected with UPEC, conferred reduced bacterial burden in the kidneys, suggesting myeloid cell-intrinsic C3aR may be inhibiting UPEC expansion during kidney infection [45]. Additionally, they found within the first 24 hours of infection, expression and secretion of inflammatory cytokines (TNF and IL-6) within the kidney and recruitment of neutrophils to the kidney are enhanced in C3aR^−/−^ mice, compared to WT [45]. Thus, this group demonstrated a protective role of C3aR via dampening the host inflammatory response to UPEC infection of the kidneys.

When defining the mechanism of C3aR-dependent suppression of inflammation and UPEC expansion, they showed *in vitro* C3a inhibited TNF and IL-6 expression in LPS-treated peritoneal macrophages, which correlated with decreased phosphorylation of ERK1/2 and IκB [45]. It was not tested if UPEC mediated the same C3a-dependent inflammatory signaling response. These data are in opposition to inflammatory data described previously by our lab, that demonstrate C3aR^−/−^ macrophages isolated from the bone marrow of mice and primary human macrophages treated with a C3aR inhibitor exhibit significantly less IL-6 and TNF when challenged with LPS [11]. These conflicting results may be due to the difference in macrophage subclass. Further, they found that C3aR^−/−^ peritoneal phagocytes (neutrophils, monocytes, and macrophages) had an impaired capacity to phagocytose UPEC and C3aR^−/−^ mice infected with UPEC showed decreased bacterial association with phagocytes from the kidney [45]. These results show C3aR is required for efficient UPEC uptake by phagocytes *in vitro*, which aligns with the results determining C3aR within DCs enhances *P*. *gingivalis* phagocytosis and C3aR within neutrophils enhance*s N*. *meningitidis* phagocytosis (Fig 1) [23,39]. Mechanistic understanding of C3aR activation and how it impacts the physiology of cells will be important to define in order to understand how C3aR increases phagocytosis within multiple innate immune cell types during bacterial infection.

### C3aR reorganizes local tissues during early stages of bacterial infection

Complement is considered a serum-specific arm of the innate immune system; however, recent studies have definitively shown C3aR can be found within the phagosomes of T cells and associated with the mitochondria in epithelial cells [6,18]. Additionally, C3a has been shown to be produced by Cathepsin L within the phagosome of T cells [18]. Considering all myeloid cells can express Cathepsin L and C3 [47,48], it is hypothesized that C3a can be produced intracellularly by multiple cell types. These new studies are beginning to define the intracellular role of the C3a–C3aR axis and bolster the nonserum role of complement within specific cell types and tissues. Local tissue reprogramming by the C3a–C3aR axis during bacterial infection was best highlighted by 2 studies that explored the role of C3aR within the lungs during *Chlamydia psittaci* infection.

#### C3aR won’t let your system forget *Chlamydia*

*C*. *psittaci* is an obligate intracellular gram-negative avian pathogen that causes psittacosis in birds and can be transmitted to humans [49]. Similar to all other *Chlamydia* species, *C*. *psittaci* possess a unique developmental cycle characterized by extracellular, infectious “elementary bodies” and an intracellular, metabolically active form called “reticulate bodies” within the phagosome [50]. Importantly, chlamydial components can be recognized by TLR2, TLR4, STING, NOD1, and the NLRP3 inflammasome [51,52]. Activation of these receptors triggers cell-autonomous immunity, characterized by the activation of NF-κB and IRF3 and the subsequent release of inflammatory cytokines (IL-6, TNF, and IFNγ) [49]. Further, production of these cytokines initiates a robust adaptive immune response, characterized by both humoral and cellular immunity [49]. To evade these responses, *Chlamydia* spp. use various strategies to interfere with NF-κB activation including blocking degradation of the NF-κB inhibitor IκBα as well as preventing nuclear translocation of NF-κB [53]. Additionally, *Chlamydia* spp. employ a protease that degrades the signaling molecule TNF receptor-associated factor 3 (TRAF3) thus blocking the activation of IRF3 and subsequent induction of IFNβ in epithelial cells [54].

In mice, *C*. *psittaci* infection can be modeled by intranasal infection, and in this model, C3 is required for protection against *C*. *psittaci* infection [55]. To determine the contribution of the C3a–C3aR axis in driving C3-protection within this infection model, 2 groups found that C3aR^−/−^ mice infected with *C*. *psittaci* displayed enhanced mortality, similar to what is seen in C3^−/−^ mice [55], in comparison with WT [55,56]. Additionally, C3aR^−/−^ showed higher bacterial burden in the lungs and spleen and an increased total number of neutrophils within the BALF [56]. Surprisingly, despite higher CFUs and neutrophils within C3aR^−/−^ lungs and BALF, respectively, C3aR^−/−^ and WT had the same level of inflammatory cytokines (IL-6, TNF, or IFNγ) within the BALF throughout the duration of infection [56]. C3aR has been reported to amplify signaling cascades within various innate immune cells; thus, we suggest that they may have missed the window of time that captures the decreased inflammatory cytokine production in C3aR^−/−^ BALF, since the earliest they checked was 9 days p.i. Further, 9 days p.i. lung-draining lymph nodes (dLNs) within C3aR^−/−^ mice contained significantly fewer B cells, CD4^+^ T cells, IFNγ^+^ CD4^+^ T cells, CD8^+^ T cells, and *Chlamydia*-specific IgM and IgG [56]. Considering TLR activation is essential for priming adaptive immune responses, and C3aR enhances TLR2 and TLR4 signaling within *in vivo* and *in vitro* models, this may be the reason that the adaptive immune response in the lung-dLNs of C3aR^−/−^ mice is attenuated. Future investigation into cytokine production and innate immune cellular function at earlier time points in *C*. *psittaci* infection may provide data imperative for understanding the role of C3aR within this model system.

Building on these findings, Kohn and colleagues elegantly demonstrated that early depletion of serum-C3, via intravenous treatment with cobra venom factor (CVF) 2 days before infection in WT mice, increased disease severity and decreased recovery time, compared to mock-treated mice [55]. Additionally, late depletion of serum-C3 (6d p.i.) did not affect infection outcome, suggesting that C3 and its bioactive forms C3a and C3b, are working to enhance early innate immune function [55]. Interestingly, they confirmed that during *C*. *psittaci* lung infection, early serum-C3 depletion correlated with a partial increase in bacterial burden, but did not completely phenocopy the overwhelming increase in bacterial burden in the lungs of infected C3^−/−^ or C3aR^−/−^ mice [55]. Further, depletion of serum-C3 only conferred a minimal, and nonsignificant decrease in survival. These findings are intriguing considering CVF does not deplete intracellular C3, which has been shown to enhance cell-autonomous immunity against the intracellular bacterial pathogen *S*. *typhimurium* [57]. We suggest that the inequity of phenotypes between serum depleted C3 and the C3^−/−^ or C3aR^−/−^ mice may be due to the unidentified role of intracellular C3a/C3aR within innate immune cells.

Together, these studies demonstrate the essential role of C3aR in controlling *C*. *psittaci* infection within the lung and during dissemination [55,56]. Further, both studies confirm that C3aR is controlling the adaptive immune response to *C*. *psittaci* by manipulating innate immune cell function, including enhancing neutrophil migration to the lung and DC migration to the lung-dLNs. However, more mechanistic data describing the requirement of the C3a–C3aR axis in driving innate cell-autonomous functions during *C*. *psittaci* infection will be crucial in understanding the requirement of C3aR in resistance to *Chlamydia* spp.

## Conclusions and future directions

Overall, findings in mouse models and human samples demonstrate that C3aR is an important modulator of inflammation, innate immune cell antimicrobial function, and resistance to intracellular and extracellular bacterial pathogens (Table 1). It is clear from the conflicting results obtained thus far with C3aR^−/−^ mice and inflammation during bacterial infection that our understanding of the functional role of C3aR is limited. Additionally, there is a need to better understand how specific innate immune cell subclasses differ in their requirement for C3aR for inflammatory signaling and antimicrobial responses. Currently, there are no data using mice with cell-specific C3aR deficiencies and bacterial infection, only global C3aR knockout mice. Building these mice to investigate how C3aR modulates inflammatory responses during bacterial infection could provide mechanistic insight into how cell-specific C3aR contributes to bacterial resistance.

**Table 1:** Summary of C3aR-dependent phenotypes in bacterial infection models.

Pathogen	Gram-stain	Lifecycle	Role of C3aR *in vivo*; tissues analyzed	Role of C3aR *in vitro*; cell type analyzed	References
*Listeria monocytogenes*	+	Intracellular	Protective; Spleen, liver Anti‐inflammatory(IFNγ,IL‐6,TNF); blood	Anti‐inflammatory(IFNβ); BMDCs	[9, 34]
*Chlamydia psittaci*	-	Intracellular	Protective; Lung, liver, spleen	N/A; N/A	[55, 56]
*Neisseria meningitidis*	-	Intracellular/ Extracellular	Protective; blood Proinflammatory(IL‐6); blood	No role; murine whole blood Proinflammatory(IL‐8); human whole blood	[23]
*Porphyromonas gingivalis*	-	Intracellular/ Extracellular	N/A; N/A	Protective; BMDCs Proinflammatory(TNF,IL‐23,IL‐12p70); BMDCs No role; BMDMs	[39, 44]
Uropathogenic *E*. *coli*	-	Intracellular/ Extracellular	Protective; kidney Anti‐inflammatory(TNF,IL‐6); kidney	Anti‐inflammatory(TNF,IL‐6); peritoneal macrophages	[45]
*Pseudomonas aeruginosa*	+	Extracellular	Deleterious; lung, blood Proinflammatory(IL‐6,TNF,IL‐1β); BAL	N/A; N/A	[27]

Red font signifies an inflammatory role for C3aR; blue font signifies an anti-inflammatory role for C3aR.

Further, relatively new studies have identified an intracellular role for C3aR. Intraphagosomal production of C3a via cleavage of phagosomal C3 by hydrolytic enzyme Cathepsin L has been shown to regulate T cell homeostasis and the response to antigens [18]. Additionally, a recent study has shown that C3aR can localize to the mitochondrial membrane within epithelial cells and become activated by intracellular C3a during H_2_O_2_-induced oxidative stress [6]. Together, these recent studies conclude intracellular production of C3a and C3aR activation regulates homeostatic and stress responses in various cell types. Thus, it is imperative that we revisit studies looking at C3aR-mediated bacterial resistance with the new understanding that C3a may be coming from within the cell, and not from the serum.

Importantly, there is recent evidence showing that infection of respiratory epithelial cells with Severe Acute Respiratory Syndrome Coronavirus 2 (SARS-CoV-2) leads to significant induction of the complement system [58,59]. Specifically, SARS-CoV-2 infection of respiratory epithelial cells generated secreted C3a that acted on bystander immune cells such as macrophages to drive a hyperactivation state that tracked with disease severity in patients with Coronavirus Disease 2019 (COVID-19) [58]. This evidence highlights the need to better distinguish the role of myeloid cell-intrinsic C3aR activation versus bystander C3aR activation, how crosstalk between immune and nonimmune cells contributes to complement hyperactivation, and the signaling events downstream of C3aR involved in promoting pathological complement activation during viral and bacterial infections. Additionally, understanding the role of C3aR in regulating secondary bacterial infections during viral infections, a clinical manifestation common in pandemic respiratory viruses such as SARS-CoV-2 [58–60], may prove beneficial to developing therapeutics for future viral pandemics. Lastly, with the recent evidence of C3aR enhancing antigen uptake and T-cell stimulation in DCs and its effect on phagocytic capacity in phagocytes, future studies on how C3aR primes the immune system for better adaptive responses may be useful in developing better vaccines and combating infectious diseases.
